# GIT1 gene deletion delays chondrocyte differentiation and healing of tibial plateau fracture through suppressing proliferation and apoptosis of chondrocyte

**DOI:** 10.1186/s12891-017-1653-7

**Published:** 2017-07-28

**Authors:** Peng Chen, Wan-Li Gu, Ming-Zhi Gong, Jun Wang, Dong-Qing Li

**Affiliations:** 1 Department of Trauma Orthopedics, The Second Hospital of Shandong University, Jinan, 250033 People’s Republic of China; 2 Department of Operating Theater, The Second Hospital of Shandong University, No. 247, Beiyuan Street, Jinan, 250033 Shandong Province People’s Republic of China

**Keywords:** GIT1, Gene deletion, Tibial plateau fracture, Proliferation, Apoptosis

## Abstract

**Background:**

Although tibial plateau fracture is an uncommon injury, its regulation is challenging and there are some influencing factors, including the effects of severe bone displacement, depression and cancellous bone cartilage, and inevitable cartilage damage. And GIT1 plays an important role in bone mass and 78 osteoblast cell migration.

**Methods:**

The study used 72 C57/BL6 mice. A tibial plateau fracture model was established by using mice with the same number of GIT1 gene deletions (the experimental group) and their wild-type littermates (the control group). Joint and bone callus recovery were evaluated by X-ray and CT thin layer scans. Micro CT assay and histomorphometry were conducted in order to evaluate the volume of newly formed blood vessels. Type II collagen expression in tibial tissues after tibial plateau fracture were detected by immunohistochemistry after 7, 14 and 21 days. The number of proliferating cell nuclear antigen (PCNA) positive cells after tibial plateau fracture was tested by immunohistochemistry after 14 and 21 days. The terminal deoxynucleotidyl transferase-mediated dUTP-biotin nick end labeling (TUNEL) staining was conducted after 14 and 21 days in order to test chondrocyte apoptosis in tibial tissues after tibial plateau fracture.

**Results:**

The GIT1 gene deletion group mice spent less time on the rotating rod than the control group mice (*P* < 0.05). Compared with the control group, postoperative recovery was retarded, because GIT1 gene deletion slowed down neovascularization after tibial plateau fracture (*P* < 0.05). Compared with the control group, mouse type II collagen expression significantly decreased in the GIT1 gene deletion group, and the proportion of PCNA positive cells significantly decreased (*P* < 0.05). The TUNEL results indicate that GIT1 gene deletion led to reduced chondrocyte apoptosis.

**Conclusion:**

GIT1 gene deletion can inhibit chondrocyte proliferation and apoptosis during the recovery of tibial plateau fracture, so as to delay chondrocyte differentiation and tibial plateau fracture healing.

## Background

Tibial plateau fractures are thought of as uncommon injuries, and are not frequently reported in literature [1]. Tibial plateau fractures are composed of a large spectrum of fracture types, and occur due to a combination of axial load and forces of valgus that are severe and difficult to treat [2, 3]. The regulation of tibial plateau fractures is challenging and there are some influencing factors, including the effects of severe bone displacement, depression and cancellous bone cartilage, and inevitable cartilage damage [4]. The nature of the tibial plateau fractures determines anatomical reduction, tibial localization, and stable fixation, which could promote bone healing and reduce the risk of traumatic osteoarthritis [5]. Some previous studies investigated that some medical treatments such as rivaroxaban and bisphosphonates, exert positive effects on fracture healing [6, 7]. In addition, open reduction and internal fixation is regarded as the best treatment for tibial plateau fractures, but soft tissue injury, knee instability, meniscal damage and possible treatment of compartment syndrome can possibly affect the treatment [8, 9]. The high risk of thromboembolic complications exists in patients with tibial plateau fractures, wherein high energy trauma is considered to be a major cause of poor outcomes of treatments [3, 10]. In order to prevent traumatic osteoarthritis and help recover joint functioning, congruency of the articular surface, stability, and correct load distribution need to be completly restored [11].

The G-protein coupled receptor (GPCR)-kinase interacting proteins 1 (GIT1) belongs to the GIT subfamily of ADP ribosylation factor (ARF)-GTPase including GIT1 and GIT2, which could stimulate GTPase activity of proteins [12]. The GIT1 gene is located on chromosome 17p11.2, and it encodes a broad multi-domain protein involved in a lot of cellular processes [13]. Also, GIT1 is capable of interacting with many proteins and producing protein-protein interaction networks, and consequently, could regulate spine and synapse development [14]. GIT1 establishes control of a variety of cellular functions and is highly expressed in neurons, endothelial cells and vascular smooth muscle cells (VSMC) [12]. A previous study proved that the deletion of the ARF-GAP domain of the GIT1 gene could contribute to the inhibition of neutral growth [13]. Besides, GIT1 is a prognostic biomarker and could promote tumor progression, which is known to induce adhesion formation and cell migration [15]. GIT1 plays an important role in bone mass and osteoblast cell migration, in which the structure of GIT1 protein could adhere to osteoblasts by inhibiting P-ERK1/2 [16, 17]. Therefore, we hypothesize that GIT1 gene deletion may delay differentiation of chondrocyte and healing of tibial plateau fracture through suppressing proliferation and apoptosis of chondrocyte.

## Methods

### Study subjects

A total of 36 GIT1 knockout mice with C57/BL6 background were purchased from Nanjing Medical University, and the GIT1 heterozygous mice were obtained after more than seven backcross generations. The GIT1 homozygous knockout mice were obtained by interbreeding GIT1 heterozygous mice with C57/BL6 background. A total of 36 wild-type mice of littermates were adopted as the control group. All mice were kept in a clean environment at a constant temperature of 22 °C and 49.7% humidity. The specific information of mice was presented as follow (Table 1).

**Table 1:** Message of mice

	GIT1-WT	GIT1-KO
Strain	C57BL/6	C57BL/6
Age (weeks)	8	8
Sex	Male (16) Female (20)	Male (17) Female (19)
Weight (g)	Male(23.3 ± 2.0g) Female (21.1 ± 1.8 g)	Male (22.4 ± 1.9 g) Female (20.3 ± 2.0 g)
Quantity	36	36

### Model establishment and rotarod test

Establishment of the tibial plateau fracture model was as follows: mice were anesthetized using 10% hydrated chlorine intraperitoneal injections and their legs were disinfected by washing with 75% alcohol. A short incision was made from the outside of the mouse thigh to the joint; followed by a long incision in the patellar tendon and a 0.5 mm hole was drilled in the tuberositas tibiae. A 25 G needle was placed inside the tibial bone marrow cavity. Subsequently, the wound was sutured. Mice were immediately undertaken weight-bearing after awakening. Mice bones were injected with Gentamicin (Sangon Biotech Co., Ltd. Shanghai, China) to prevent infections for the initial 3 days after surgery. 12 mice were sacrificed on day 7 and day 14 after surgery, respectively. 12 mice were sacrificed for follow-up experiments on day 21 after surgery. The remaining 12 mice in each group were trained on a rotating rod at a speed of 20 r/min, and training was conducted twice a day for 10 mins on day 18 after surgical procedures, once in the morning and once in the evening. Once the mice were trained up to day 20, six mice capable of staying on the rotating rod for more than 40 s were selected from each group (if more than six mice were competent, mice would be selected randomly). On day 21, the selected mice were placed on the rotating rod at the speed of 20 r/min. The duration of time since the mice were placed on the rotating rod till dropping out was recorded.

### X-ray and Micro CT assay

X-ray and CT thin layer scanning were used to observe joint recovery after tibial plateau fractures. CT thin layer scanning was as follows: mice tibias were taken out at the appropriate time and subsequently scanned using a Viva Micro CT system at a voxel size of 10.5 μm (Scanco Medical AG, Basserdorf, Switzerland). A radiopaque silicone rubber compound containing lead chromate was perfused via the heart along with 4% paraformaldehyde following an initial vascular flush with heparinized saline for angiography. At that point the heart was washed with phosphate-buffered saline (PBS) containing heparin. The fractured part of the tibia was taken out and decalcified with 10% ethylenediaminetetraacetic acid (EDTA) for 21 days. Micro CT was conducted to scan blood vessel.

### Immunohistochemistry

The extracted tibial tissues of mice were fixed in a 10% formaldehyde solution, decalcified with EDTA, embedded in paraffin, and sectioned. After that, the parafin sections of tibial tissues were selected on the day 7, day 14 and day 21 and dewaxed and hydrated using xylene and graded alcohol, respectively. Post hydration, the sections were penetrated with 0.2% Triton X-100 PBS for 10 min, washed with PBS, and blocked with PBS containing 3% goat serum for 30 min. Subsequently, primary antibody, type II collagen (Collagen II, 1:200, Sigma) was added into the sections followed by overnight incubation at 4 °C. The next day, post PBS wash, biotin secondary antibody (Zymed Company, San Diego, USA) was added to the sections and incubated for 30 min. PBS was used for washing the sections, and then ABC reagent (Zymed Company, San Diego, USA) was dripped into the sections. After 30 mins incubation, the sections were observed under a microscope, and semi-quantitative analysis was adopted in order to observe staining area of type II collagen using Image-Proplus 5.02 image analysis system.

The parafin sections of tibial tissues on day 14 and day 21 were selected for conducting proliferating cell nuclear antigen (PCNA) immunohistochemistry. Mice PCNA monoclonal antibody (Zymed) was sectioned, dewaxed routinely and hydrated, followed by tissue antigen repair with the help of a microwave for 5 min (citrate buffer solution was used as repair fluid, pH 6.0). Next, the sections were washed by PBS after room temperature incubation for 10 min in 30% H_2_O_2_. The rabbit primary antibody (1:100, Wuhan Boster Biological Technology, Co. Ltd., Wuhan, China) was added after 4 °C overnight incubation with PCNA (dilution 1: 200, Invitrogen, CA, USA). Next, the sections were washed with PBS, followed by the addition of goat secondary antibody (1:100, Wuhan Boster Biological Technology, Co. Ltd., Wuhan, China) for incubation another time at room temperature for 30 min. The sections were washed again with PBS, developing with DAB for 5 min, staining with haematoxylin, dehydration with gradient alcohol, Xylene transparent and mounting with neutral resin. Subsequently, the sections were observed under a microscope.

### TDT-mediated dUTP-biotin nick end-labeling (TUNEL)

Paraffin sections of tibial tissues were dewaxed and hydrated following the aforementioned methods on day 14 and day 21, and then the sections were penetrated with 0.1% Triton X-100. After the addition of TUNEL reaction complexes (In Situ Cell Death test kits, MultiSciences (Lianke) Biotech Co., Ltd., Hangzhou, China), tibial tissue sections were incubated for 1 h in a cell incubator. Subsequently, DAPI staining was employed for 5 min in order to label the cells. Tibial tissue sections were observed under a fluorescence microscope (Olympus, Tokyo, Japan), and post observation, the TUNEL positive cells were considered as apoptotic cells.

### Statistical analysis

Statistical analysis was performed using the SPSS 18.0 statistical software. Measurement data were expressed as mean ± standard deviation (SD). Quantile-quantile plot was applied to analyze statistical data in each group, and the data was in accordance with normal distribution of small residuals. A two-tailed unpaired *t* test was applied for comparisons between two groups. The modeling curve was adopted for kinetic analysis of angiogenesis after surgery. *P* < 0.05 was considered as statistically significant.

## Results

### Behavioral observation of mice and results of rotarod test

**Fig. 1:**
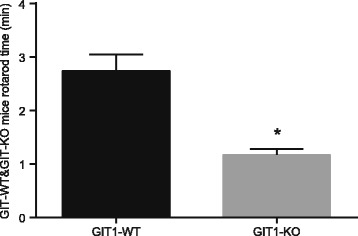
The results of the rotarod test in the GIT1-WT and GIT1-KO group. Note: *, *P* < 0.05, compared with the control group

On the 14^th^ day after operation, mice in the control group were less active in the cage, and right fracture hind limbs of these mice were raised and were unable to touch the ground without external help, while mice in the experimental group were almost inactive in the cage. On the 21st day after operation, mice in the control group showed an increase their activities, and a majority of the control mice elevated the fracture sides of their bodies. Individual mice in this group could slightly touch the ground with their fracture sides. Whereas, mice in the experimental group scarcely began to move in the cages, they were unable to touch the ground with their fracture hind limbs. Meanwhile, the results of rotarod test (Fig. 1) showed that the experimental group mice spent less time on the rotating rod in comparison to the control group mice (*P* < 0.05), which indicates that postoperative recovery after tibial plateau fracture of mice in experimental group was slower than in control group.

### Effects of GIT1 gene deletion on recovery of joint function after tibial plateau fracture

**Fig. 2:**
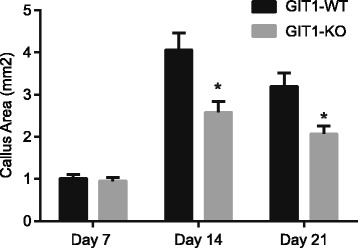
The results of CT thin layer scans of tibial plateau in the control and experiment groups on the 14th day after operation. Notes: *P < 0.05, compared with the control group; CT: Computed tomography

In order to observe the effect of GIT1 gene deletion on recovery of joint function after tibial plateau fracture, both mice groups were subjected to total body X-ray irradiation on day 14 after operation. The tibial plateau of mice in the control group began healing on the 14^th^ day after operation, while the healing process of mice in the experimental group was evidently delayed. In order to further evaluate the fracture healing in the two groups, the CT thin layer scanning was employed and findings indicate that bone callus in the GIT1 gene deletion group (the experimental group) was significantly less than that in the control group (Fig. 2). Meanwhile, on the 7^th^ day, there was no significant difference in the area of bone callus between the two groups, while on the 14^th^ day and the 21^st^ day, the area of bone callus in experimental group was significantly less than in the control group. The findings indicate that GIT1 gene deletion would result in delayed recovery of tibial plateau fracture.

### Effects of GIT1 gene deletion on neovascularization after tibial plateau fracture

**Fig. 3:**
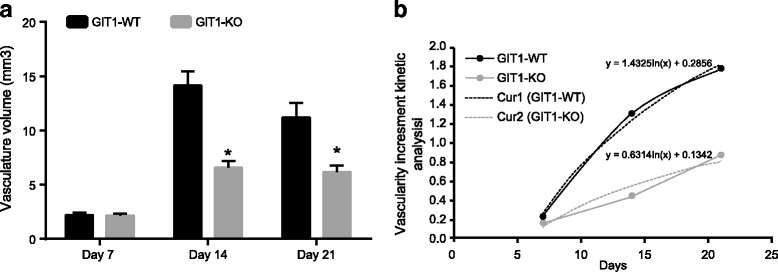
Effects of GIT1 gene deletion on neovascularization after tibial plateau fracture. Notes: **a**, volume statistics of new blood vessels; **b**, kinetic analysis of the blood vessels growth after operation; Cur1, modeling curve of statistical curve in the GIT1-WT group (coefficient 1.432); Cur2, modeling curve of statistical curve in the GIT1-KO group (coefficient 0.631); *, *P* < 0.05, compared with the control group

The effects of GIT1 gene deletion on neovascularization after tibial plateau fracture are similar to the effects of GIT1 gene deletion on healing after operation. On the 14th day, the new vascular volume of mice in the experimental group was less than 50% of the control group, and on the 21st day, the new vascular volume of mice in the experimental group was less than 60% of the control group (Fig. 3). Kinetic analysis was applied in order to analyze the growth of blood vessels. After model establishment, the speed of increasing blood vessels of GIT1-WT group was more than twice of GIT1-KO group, which indicates that GIT1 gene deletion results in inhibited vascular invasion and delayed new bone formation in GIT1-KO group, which culminated in delayed postoperative recovery after tibial plateau fracture.

### Effects of GIT1 gene deletion on endochondral bone formation after tibial plateau fracture

**Fig. 4:**
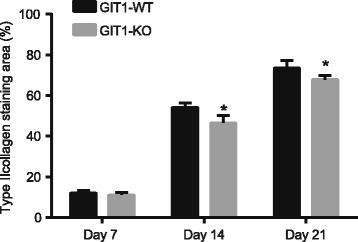
Comparisons of immunohistochemistry results of type II collagen in tibial tissues between the control and experimental groups. Notes: *, *P* < 0.05, compared with the control group

Type II collagen expression was a marker for cartilage progenitor cells differentiating into chondrocytes, which represents the existence of chondrocytes. 7 days after tibial plateau fracture, the expression of type II collagen was similar between the control group and experimental group mice (Fig. 4). While on 14^th^ day and the 21^st^ day after operation, the expression of type II collagen of mice in the experimental group was significantly higher than the control group. These results suggest that GIT1 gene deletion would result in delayed chondrocyte differentiation during the process of fracture healing.

### Effects of GIT1 gene deletion on proliferation of chondrocytes after tibial plateau fracture

**Fig. 5:**
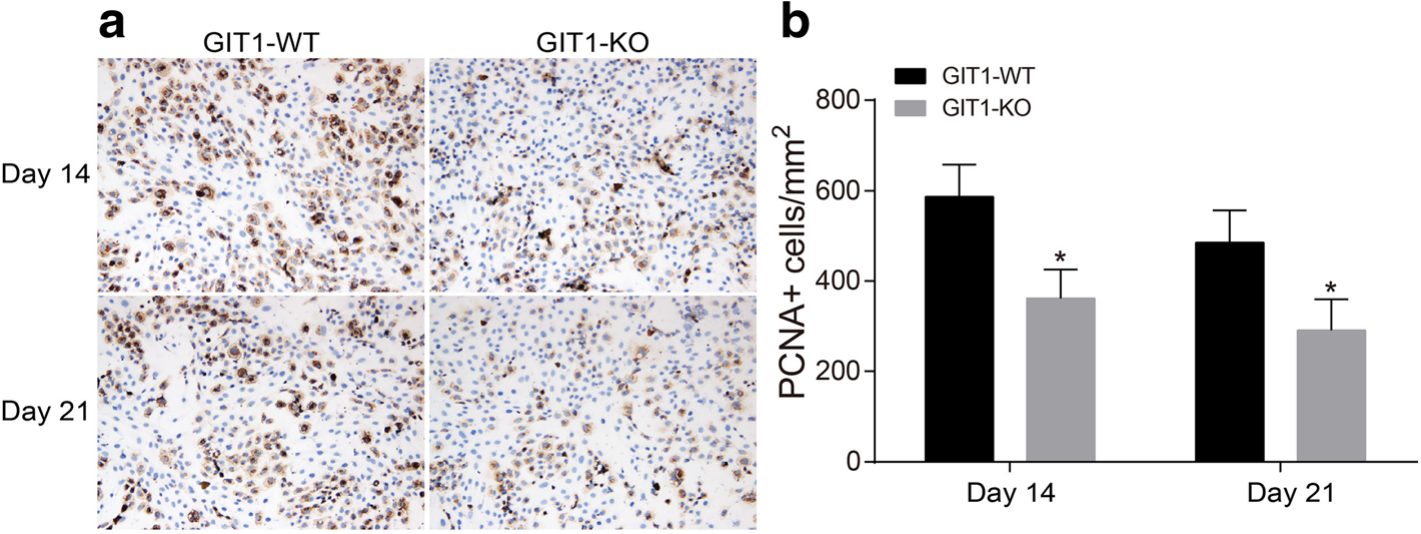
Comparison of immunohistochemistry results of PCNA staining in tibial tissues between the control and experimental groups. Notes: **a** immunohistochemistry results of PCNA staining in tibial tissues (×400); **b** the number of PCNA positive cells in each bone callus; *, *P* < 0.05, compared with the control group; PCNA: Proliferating cell nuclear antigen

The delay in chondrocyte differentiation may result from the decreased chondrocyte proliferation. To identify the effect of GIT1 gene deletion on chondrocyte proliferation in the recovery process of tibial plateau fracture, PCNA antibody staining was conducted to indicate cell proliferation (Fig. 5). Compared with the control group, PCNA positive cells in the bone callus were significantly decreased in the experimental group (*P* < 0.05). The results indicate that GIT1 gene deletion could inhibit chondrocyte proliferation.

### Effects of GIT1 gene deletion on apoptosis of chondrocyte after tibial plateau fracture

**Fig. 6:**
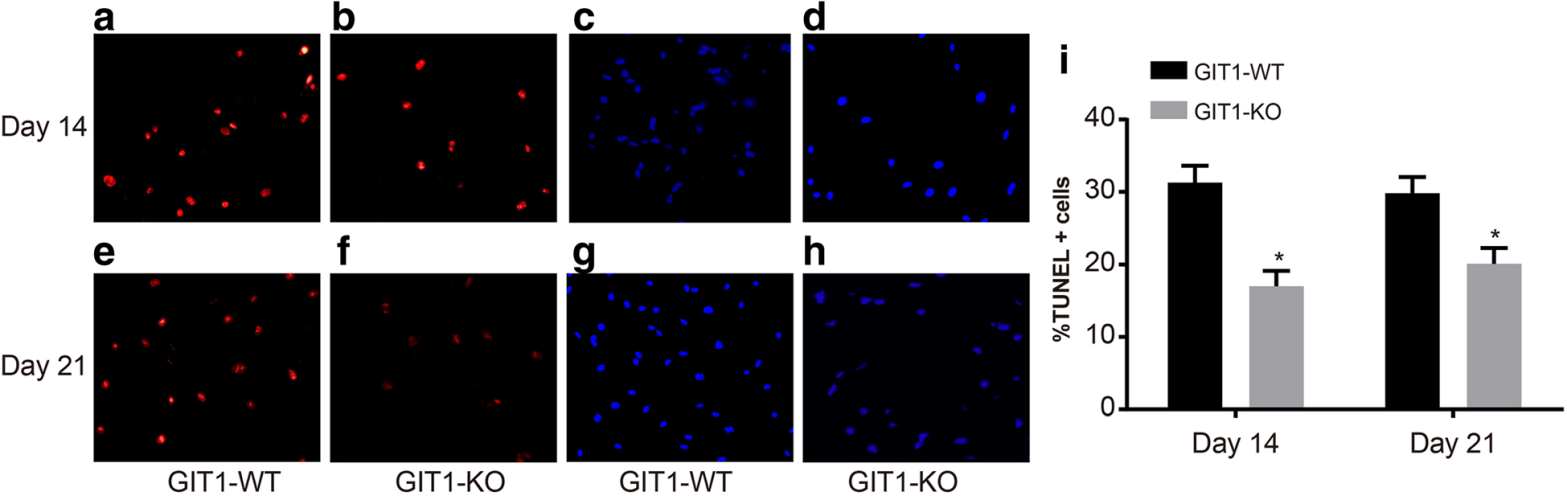
Effects of GIT1 gene deletion on apoptosis of chondrocytes after tibial plateau fracture. Notes: **a**, **b** TUNEL staining result on the 14^th^ day after surgery (×200); **c** **d**, DAPI staining result on the 14^th^ day after surgery (×200); **e**, **f** TUNEL staining result on the 21st day after surgery (×200); **g** **h**, DAPI staining result on the 21st day after surgery (×200); **i**, The number of TUNEL positive cells (TUNEL positive cells/DAPI positive cells); *, *P* < 0.05, compared with the control group; DAPI: Diamidino-phenyl-indole; TUNEL: TDT-mediated dUTP-biotin nick end-labeling

The persistent presence of chondrocyte may be associated with decreased chondrocyte apoptosis during the recovery process of tibial plateau fracture. TUNEL staining was used in order to assess GIT1 gene deletion effects on apoptosis of chondrocyte (Fig. 6). Compared with mice in the control group, the number of TUNEL positive cells on day 14 and day 21 after operation in experimental group mice was significantly decreased. DAPI staining was adopted to mark the number of cells, followed by calculation of the percentage of TUNEL positive cells. The results show that the percentage of TUNEL positive cells was decreased in experimental group mice (*P* < 0.05).

## Discussion

Fracture healing is a poorly understood process, wherein osteoblast migration and proliferation are the fundamental bone healing processes, and GIT1 gene is a key regulator of bone mass and osteoblast cell migration [17, 18] Furthermore, GIT1 is the key to fracture healing, and a lot of key amino acids could influence fracture healing after GIT1 gene deletion [19, 20]. Therefore, our study aims to explore the underlying mechanism of GIT1 and tibial plateau facture and chondrocyte cells.

Our study found that GIT1 gene deletion could block the healing of tibial plateau fractures, wherein chondrocytes exist persistently and delay cell differentiation. Fracture healing is a process of post natal repair, and many molecular mechanisms could control cell differentiation and embryo development [21]. Consistently, chondrocytes play an essential role in the process of repairing osteoarthritis [22]. It is well-known fact that vascular endothelial growth factor (VEGF) plays a crucial role during endochondral bone formation in hypertrophic cartilage remodeling, ossification, and angiogenesis [23]. In addition, a previous study showed that the increase of bone mass of GIT1 wild type control GIT1 knockout (KO) mice is 2.3 times that of wild type GIT1 knockout (KO) mice control, causing bone defects, and expression of GIT1 could be found in osteoclast and chondrocyte cells [18]. Similarly, a variety of growth factors have been used in order to accelerate the healing process including platelet-derived growth factor (PDGF) and TGF-β, and PDGF could regulate chondrocyte proliferation through the activation of GIT1, which proves that GIT1 gene deletion could delay the healing process [24, 25]. Moreover, a previous study indicated that differentiation and maturation of chondrocytes are crucial in fracture healing, involved in the entire process of fracture healing, which is consistent with our study results [26]. Likewise, GIT1 deficiency impairs osteoclast functioning, and is required for osteoclasts differentiation, which could prove our results [18]. Additionally, GIT1 is capable of regulating fracture healing through the TGF-β signaling pathway, and previous studies confirmed that TGF-β could inhibit cell growth by controlling chondrocyte synthesis and promoting the differentiation process of the chondrocytes [20, 27], so GIT1 gene deletion is capable of delaying chondrocyte differentiation. Besides, GIT1 gene deletion could regulate the fracture healing process, and findings suggest that chondrocytes differentiation is affected by the loss of GIT1 [28].

Additionally, GIT1 gene deletion could contribute to the decreasing chondrocyte proliferation and apoptosis after tibial plateau fracture. Furthermore, a major function of GIT1 is to regulate cytoskeletal dynamics to facilitate cell spreading and spatial targeting, and GIT1 is a potential target for the treatment of osteoporosis [17, 18]. In addition, a number of growth factors such as PDGF could promote the proliferation of chondrocytes effectively, indicating that GIT1 gene deletion could inhibit cell proliferation [24]. Similarly, a study found that GIT1 is involved in the normal progression of bone injury after repair, and GIT1 gene KO mice could also decrease chondrocytes proliferation and inhibit apoptosis, which is consistent with our results [28]. Moreover, integrin-β1 and GIT1 gene are positively correlated, and integrin-β1 has the ability to regulate chondrocyte proliferation and apoptosis, demonstrating that GIT1 gene deletion could significantly decrease chondrocyte apoptosis [22].

## Conclusion

In conclusion, this study explored the relations between GIT1 gene deletion and joint function recovery and chondrocyte differentiation. GIT1 gene deletion could delay chondrocytes differentiation and tibial plateau fracture healing by suppressing chondrocytes proliferation and apoptosis, and may become a new target for the treatment of endothelial cell injury.

## Abbreviations

ARF: ADP ribosylation factor
EDTA: ethylenediaminetetraacetic acid
GIT1: G-kinase interacting proteins 1
GPCR: G-protein coupled receptor
KO: knockout
PBS: phosphate-buffered saline
PCNA: proliferating cell nuclear antigen
PDGF: platelet-derived growth factor
SD: standard deviation
TUNEL: TDT-mediated dUTP-biotin nick end-labeling
TUNEL: transferase-mediated dUTP-biotin nick end labeling
VEGF: vascular endothelial growth factor
VSMC: vascular smooth muscle cells
